# Validation of the International IgA Nephropathy Prediction Tool in the Greek Registry of IgA Nephropathy

**DOI:** 10.3389/fmed.2022.778464

**Published:** 2022-02-15

**Authors:** Marios Papasotiriou, Maria Stangou, Dimitris Chlorogiannis, Smaragdi Marinaki, Dimitrios Xydakis, Erasmia Sampani, Georgios Lioulios, Eleni Kapsia, Synodi Zerbala, Maria Koukoulaki, Georgios Moustakas, Stavros Fokas, Evangelia Dounousi, Anila Duni, Antonia Papadaki, Nikolaos Damianakis, Dimitra Bacharaki, Kostas Stylianou, Hariklia Gakiopoulou, George Liapis, Georgios Sakellaropoulos, Evangelos Papachristou, Ioannis Boletis, Aikaterini Papagianni, Dimitrios S. Goumenos

**Affiliations:** ^1^Department of Nephrology and Kidney Transplantation, University Hospital of Patras, Patras, Greece; ^2^Department of Nephrology, Hippokration General Hospital, Aristotle University of Thassaloniki, Thessaloniki, Greece; ^3^Department of Medical Physics and Bioinformatics, University of Patras, Patras, Greece; ^4^Department of Nephrology and Kidney Transplantation, Laiko General Hospital of Athens, National and Kapodistrian University of Athens Medical School, Athens, Greece; ^5^Department of Nephrology, General Hospital of Heraklion “Venizeleio-Pananeio”, Heraklion, Greece; ^6^Department of Nephrology, General Hospital of Nikaia, Piraeus, Greece; ^7^Department of Nephrology, Gennimatas General Hospital of Athens, Athens, Greece; ^8^Department of Nephrology, School of Medicine, University of Ioannina, Ioannina, Greece; ^9^Department of Nephrology, General Hospital of Chania, Chania, Greece; ^10^Second Department of Internal Medicine-Propaedeutic, Research Institute and Diabetes Centre, Attikon University Hospital, National and Kapodistrian University of Athens Medical School, Athens, Greece; ^11^Department of Nephrology, University of Crete, Heraklion, Greece; ^12^First Department of Pathology, National and Kapodistrian University of Athens Medical School, Athens, Greece

**Keywords:** IgAN prediction tool, IgAN disease progression, chronic kidney disease, immunosuppression, ACE inhibitors

## Abstract

**Background:**

Immunoglobulin A nephropathy (IgAN) is among the commonest glomerulonephritides in Greece and an important cause of end-stage kidney disease (ESKD) with an insidious chronic course. Thus, the recently published International IgAN prediction tool could potentially provide valuable risk stratification and guide the appropriate treatment module. This study aimed to externally validate this prediction tool using a patient cohort from the IgAN registry of the Greek Society of Nephrology.

**Methods:**

We validated the predictive performance of the two full models (with or without race) derived from the International IgAN Prediction Tool study in the Greek Society of Nephrology registry of patients with IgAN using external validation of survival prediction models (Royston and Altman). The discrimination and calibration of the models were tested using the C-statistics and stratified analysis, coefficient of determination (${\text{R}}_{\text{D}}^{2}$) for model fit, and the regression coefficient of the linear predictor (β_PI_), respectively.

**Results:**

The study included 264 patients with a median age of 39 (30–51) years where 65.2% are men. All patients were of Caucasian origin. The 5-year risk of the primary outcome (50% reduction in estimated glomerular filtration rate or ESKD) was 8%. The ${\text{R}}_{\text{D}}^{2}$ for the full models with and without race when applied to our cohort was 39 and 35%, respectively, and both were higher than the reported ${\text{R}}_{\text{D}}^{2}$ for the models applied to the original validation cohorts (26.3, 25.3, and 35.3%, respectively). Harrel's C statistic for the full model with race was 0.71, and for the model without race was 0.70. Renal survival curves in the subgroups (<16th, ~16 to <50th, ~50 to <84th, and >84th percentiles of linear predictor) showed adequate separation. However, the calibration proved not to be acceptable for both the models, and the risk probability was overestimated by the model.

**Conclusions:**

The two full models with or without race were shown to accurately distinguish the highest and higher risk patients from patients with low and intermediate risk for disease progression in the Greek registry of IgAN.

## Introduction

Immunoglobulin A nephropathy (IgAN) is considered to be the most frequent biopsy and proven type of glomerulonephritis with an estimated incidence of more than 1.5 per 100,000 persons every year. It can cause end-stage kidney disease (ESKD), in most instances, after a median disease course of more than 10 years (1). A particular feature of IgAN is the heterogeneous risk of progressive kidney function deterioration, with a 10-year risk of ESKD between 5 and 60%. Thus, IgAN treatment can be challenging, although Kidney Disease Improving Global Outcomes (KDIGO) guidelines recommend risk stratifying patients so that immunosuppressive treatment can be targeted to those at high-risk for disease progression; this stratification is based only on the degree of proteinuria which can be highly inaccurate. Until recently, there was no other tool available to accurately predict kidney disease progression (2). Nevertheless, a proportion of patients who presented with proteinuria of more than 1 g/day, and according to guidelines should be treated with immunosuppression therapy, have non-progressive disease. On the contrary, many patients with lower-grade proteinuria, who do not qualify for treatment, experience progressive disease (3, 4). This points out the necessity for an accurate clinical tool that predicts disease progression in IgAN.

Although there are well-accepted clinical and histological risk factors for kidney disease progression in IgAN, when these factors are used individually, they are unable, in many cases, to identify high-risk patients (3). Attempts in the past to establish a prediction model have not met widespread acceptance (5, 6). Although the Oxford MEST [mesangial (M) and endocapillary (E) hypercellularity, segmental sclerosis (S), and interstitial fibrosis/tubular atrophy (T)] histologic score in IgAN has been validated in international patient cohorts and is independently associated with a higher or lower risk of kidney function deterioration, it has only been recently incorporated into a risk prediction tool (7). This tool was developed by the International IgA Nephropathy Network, and it validated two versions: the full model without and with race. Although this tool has been validated and proved accurate in international cohorts of multi-ethnicity patients, there is still a paucity of evidence for its accuracy in single ethnic groups. Thus, this study aimed to validate the International IgA Nephropathy Prediction Tool using a large ethnic-based contemporary data set of patients with IgAN who were from Greece with fully available clinical, laboratory, and histological data.

## Methods

### Patients

In this study, we included patients from the Greek Society of Nephrology IgAN registry (8). In this registry, patients with biopsy-proven IgAN are reported independently for research purposes from different nephrology departments across Greece. This cohort currently consists of 657 patients. Of these, only patients with available MEST scores and estimated glomerular filtration rate (eGFR) data with long-term follow-up after biopsy (over 1 year) were included in the final analysis. Furthermore, we included only those who were 18 years or older and who did not have established ESKD at the time of biopsy. This project was approved by the research ethics committee of the University Hospital of Patras, which waived patient's written informed consent for using their anonymized historical clinical data.

### Definitions

Age, proteinuria, eGFR (using the Chronic Kidney Disease Epidemiology Collaboration formula), systolic blood pressure (SBP), diastolic blood pressure (DBP), mean arterial blood pressure (MAP = 1/3 x SBP + 2/3 x DBP), body mass index (BMI), prior use of medications that block the renin-angiotensin system blockers (RASBs, including angiotensin-converting enzyme inhibitors and angiotensin receptor blockers), and the use of immunosuppression were determined at the time of biopsy and during follow-up. The decline slope of eGFR was calculated using a linear regression line.

All patients included in this cohort were white Caucasians. Kidney biopsies were scored according to the Oxford MEST scoring system (Supplementary Figure 1) at the time of diagnosis by three pathologists who were not blinded to clinical data as a standard procedure (9). Crescent formation in kidney biopsies (C score) was not incorporated in the prediction tool, as according to the International IgAN tool proposed by Barbour et al. in the original derivation cohort, this variable did not correlate significantly with prognosis. The primary outcome was a composite of either ESKD (eGFR <15 mL/min/1.73 m^2^, dialysis or kidney transplantation) or a reduction in eGFR below 50% of the value at biopsy for a period of more than 3 months, whichever occurred first. For validation, each covariate and outcome were defined exactly according to the original publication of Barbour et al. (10).

### Prediction Models for External Validation

The prediction models for external validation were derived from the original publication of Barbour et al. (10) and described in detail by Zhang et al. (11).

### Statistical Analysis

The initial step for the model validation was to calculate the linear predictor for each patient in our cohort based on the exact predictors and coefficient values. Then, we assessed the discrimination and calibration performance of the model according to Royston and Altman, and Zhang et al. (11, 12).

Discrimination was assessed first by estimating the regression coefficient on the linear predictor coefficient by fitting a Cox proportional hazards model for the full model without race and an interval format Cox proportional hazards model for the full model with race in our data set. If the slope on the linear predictor is >1, then the discrimination is better, and conservatively if it is <1, the discrimination is poorer. The model parameters for the calculation of the linear predictor were taken by the original publication (10) but the linear predictor itself was calculated using the equations as described in detail by Zhang et al. (11) for each patient of our data separately.

Second, Harrell's C-index of concordance or C-statistic was calculated to determine the ability of the model to discriminate between patients who have experienced the outcome of disease progression against those who did not. By definition, the C-statistic must lie between 0.5 and 1, with a general consensus that a C-statistic with an acceptable discrimination power is ≥ 0.65. In addition, the coefficient of determination (${\text{R}}_{\text{D}}^{2}$) was also calculated using the method proposed by Royston and Sauerbrei (13).

Third, we divided the patients into risk groups, including <16th (low risk), approximately from 16 to <50th (intermediate risk), ~50th to <84th (higher risk), and > 84th (the highest risk) percentiles of the linear predictor from the full model without and with race. Subgroup analyses were performed, and survival curves were derived.

As proposed by Royston et. al., the hazard ratios can be a sensible verification of model discrimination for comparing risk groups, in contrast to the *p*-values (12). Thus, hazard ratios were evaluated by fitting a Cox model with a dummy variable representing each risk group referring to the lowest risk group. When survival curves are more widely separated, the hazard ratio tends to be greater. For model calibration, the overall estimated regression coefficient of the linear predictor (β_PI_) is the most precise estimate of relative global calibration and was calculated accordingly.

## Results

### Clinical Characteristics and Outcome of Baseline Patients

**Figure 1:**
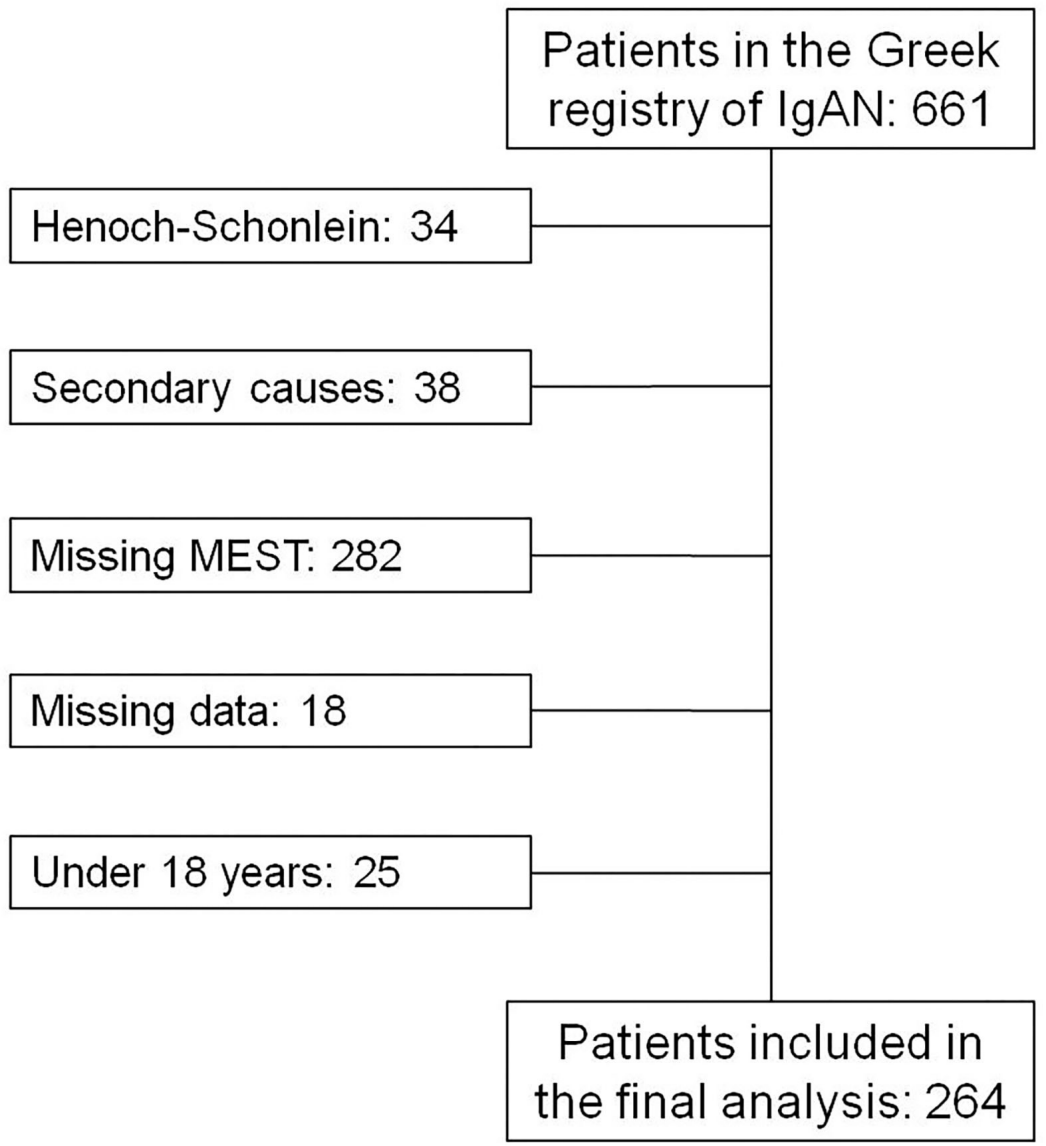
Flow chart of patients finally included in the analysis.

The flow chart of the inclusion of the patients, in the final analysis, is presented in Figure 1 and their clinical characteristics are presented in Table 1. There were 264 patients included in our analysis, all of Caucasian origin. In this cohort, the percentage of combined outcomes was 20.07% and among these, 12.9% reached ESKD and 13.6% showed a 50% decrease in eGFR. Mean follow-up was 8.5 (5–10.83) years.

**Table 1:** Comparison of clinical and histological characteristics in the current and previously reported cohorts.

**Characteristics**	**Reported derivation cohort**	**Reported validation cohort**	**This validation cohort**
Number of patients	2,781	1,146	264
Follow up (yr)	4.8 (3.0–7.6)	5.8 (3.4–8.5)	8.5 (5–10.83)
Age at biopsy (yr)	35.6 (28.2–45.4)	34.8 (26.9–45.0)	39 (30–51)
Gender (M/F %)	1,608 (57.8) / 1,173 (42.2)	565 (49.3) / 581 (50.7)	172 (65.2) / 92 (34.8)
**Race** ***n*** **(%)**			
Caucasian	1,167 (42.0)	176 (15.5)	264 (100)
Chinese	1,021 (36.7)	292 (25.8)	-
Japanese	569 (20.5)	616 (54.4)	-
Other	22 (0.8)	49 (4.3)	-
**sCr at biopsy (mmol/l)**	1.04 (0.8–1.4)	84.0 (66.2–111.4) 0.95 (0.75–1.29)	1.2 (0.9–1.775)
**eGFR at biopsy (ml/min/1.73 m** ^**2**^ **)**	83.0 (56.7–108.0)	89.7 (65.3–112.7)	61.09 (40.27–83.41)
<30, n (%)	142 (5.1)	37 (3.2)	43 (16.3)
30–60, *n* (%)	657 (23.6)	191 (16.7)	85 (32.2)
60–90, *n* (%)	800 (28.8)	350 (30.5)	83 (31.4)
>90, *n* (%)	1,182 (42.5)	568 (49.6)	53 (20.1)
**MAP (mmHg)**	96.7 (88.7–106.3)	93.3 (85.0–103.3)	100 (88.3–106.7)
**Proteinuria**			
<0.5, *n* (%)	383 (13.9)	221 (19.4)	42 (15.9)
0.5–1, *n* (%)	772 (28.1)	209 (18.3)	58 (21.9)
1–2, *n* (%)	817 (29.7)	352 (30.8)	84 (31.8)
2–3, *n* (%)	360 (13.1)	145 (12.7)	34 (12.9)
>3, *n* (%)	415 (15.1)	215 (18.8)	46 (17.4)
**MEST score**			
M1 (%)	1,054 (38.0)	481 (42.0)	186 (71)
E1 (%)	478 (17.3)	476 (41.5)	91 (34.7)
S1 (%)	2,137 (77)	912 (79.6)	154 (58.4)
T1 (%)	686 (24.7)	207 (18.1)	67 (25.6)
T2 (%)	128 (4.6)	122 (10.6)	11 (4.2)
**RASB use**, ***n*** **(%)**			
At biopsy	862 (32.4)	320 (30.0)	117 (44.5)
After biopsy	2,400 (86.7)	708 (66.4)	223 (84.5)
**Immunosuppression**			
After biopsy	1,209 (43.5)	359 (31.3)	122 (46.2)
**Primary outcome**			
50% eGFR decline	420 (15.1)	210 (18.3)	36 (13.6)
ESKD	372 (13.4)	155 (13.5)	34 (12.9)
Total primary outcome events	492 (17.7)	213 (18.6)	53 (20.07)

The rate of RASB use was 44.5% at biopsy and 84.5% after biopsy while 46.2% received a form of immunosuppressive regimen during follow-up. Immunosuppression treatment prescription according to risk for disease progression is as follows; in the lower risk group (lower 16th percentile) the immunosuppression treatment prescription was 26.2%, in the intermediate-risk group (16–50th percentile), it was 33.3%, in the higher risk group (50–84th percentile), it was 58.9%, and in the highest risk group (upper 16th percentile), it was 66.7%. The immunosuppressive treatment options and protocols that were followed by each center varied depending on local practices and experience. These included 4 main treatment protocols i.e., oral steroid treatment based on a 6-month regimen of oral prednisone starting at 1 mg/kg/day for the first 2 months and with gradual tapering until the end of treatment at 6 months which was prescribed to 57 patients (6, 20, 21, and 10 patients in the lower, intermediate, high, and highest risk groups, respectively). Another option was the Pozzi regimen consisting of i.v. bolus injections of 1 g of methylprednisolone for 3 days each at months 1, 3, and 5, followed by an oral steroid of 0.5 mg/kg prednisone on alternate days for a total of 6 months which was followed by 24 patients (5, 5, 1, and 4 patients in the lower, intermediate, high, and highest risk groups, respectively); oral steroid treatment as mentioned in the first regimen plus azathioprine 100 mg/day for 6 months was followed by 21 patients (0, 1, 11, and 9 patients in the lower, intermediate, high and highest risk groups, respectively); and finally, i.v. bolus injections of 0.75 g/m^2^ of body surface of cyclophosphamide every 4 weeks for 3 to 6 dosed in total plus 500 mg of i.v. methylprednisolone for 3 consecutive days plus oral prednisone of 1 mg/kg/day for 1 month with a maximum dose of 60 mg/day with gradual tapering over 4–6 months which was followed by 14 patients (0, 2, 8, and 4 patients in the lower, intermediate, high, and highest risk groups, respectively). Finally, another option that was scarcely used was the combination of oral prednisone with 1–2 g of mycophenolate mofetil which was followed by 6 patients (0, 2, 3, and 1 in the lower, intermediate, high, and highest risk groups, respectively). According to our data, 14 patients had IgA vasculitis and all were treated with IV cyclophosphamide. The use of immunosuppression ranged in older cohorts from 7.1 to 11.1% (11). Moreover, the distributions of other clinical parameters, including baseline kidney function, age, gender, and Oxford MEST histologic scores showed significant differences between this and previously reported studies while proteinuria and blood pressure were similar.

### Regression on Linear Predictor in Validation Data

The calibration slopes of linear prediction (β_PI_) were 0.40 for the full model without race and 0.45 for the full model with race. Thus, discrimination appeared not to be preserved.

### Measures of Discrimination and Model Fit

By applying the reported models to our current cohort, the C-statistic was calculated to be 0.70 for the model without race and 0.71 for the full model with race. In addition, $R_{D}^{2}$ values were 35% for the full model without race and 39% for the full model with race indicating an increase compared to the ~25% $R_{D}^{2}$ values of the reported derivation cohorts. Thus, according to ${\text{R}}_{\text{D}}^{2}$, and contradictory to β_PI_, a good performance of the model's fit was suggested.

### Comparison of Risk Groups

**Figure 2:**
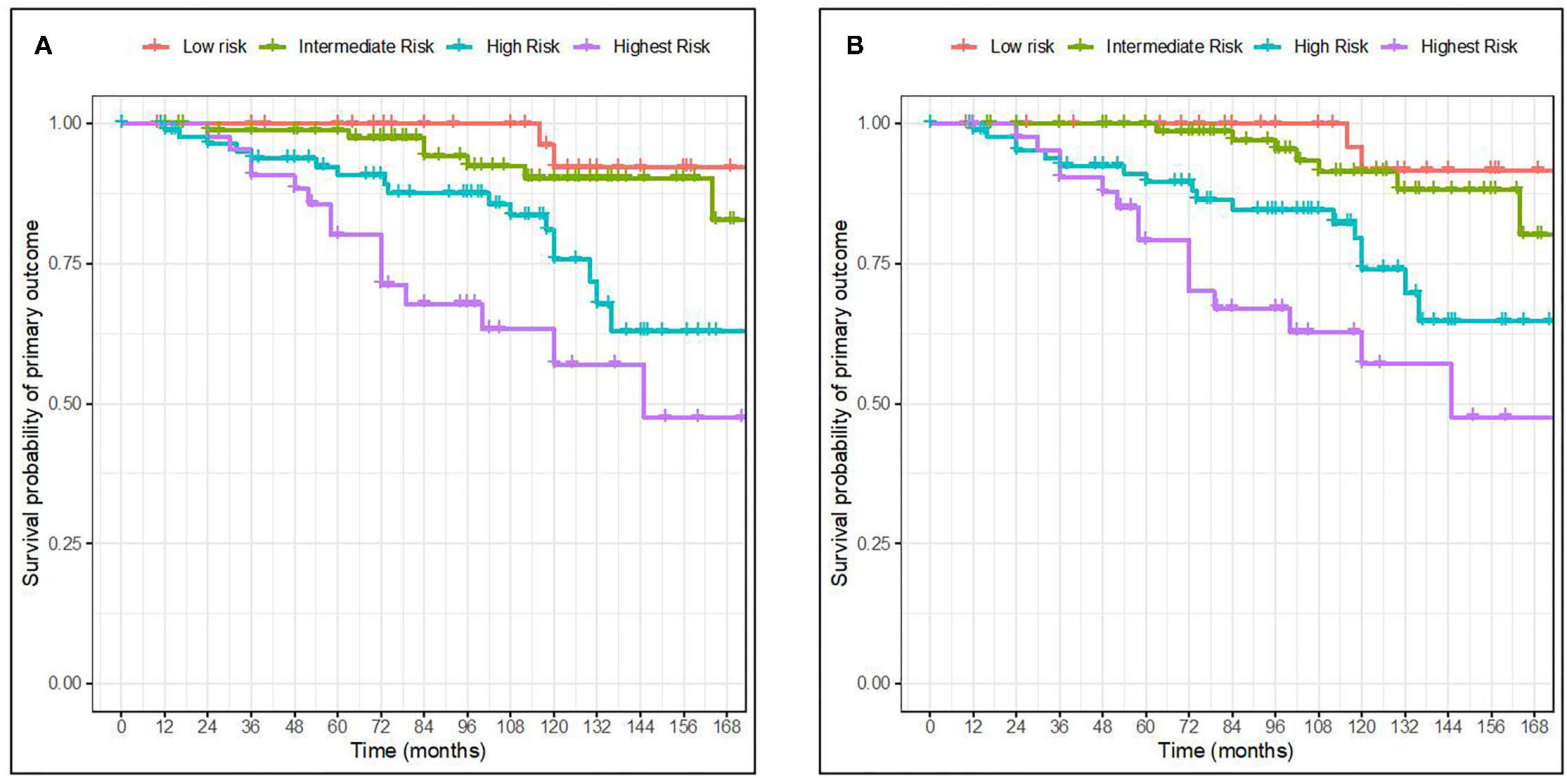
Kaplan-Meier curves for survival probability of primary outcome in 4 risk groups based on the percentile of the linear predictor. Full model with race **(A)**. Full model without race **(B)**. The 4 risk groups were defined as <16th (low risk), ~16th to <50th (intermediate risk), ~50th to <84th (higher risk), and >84th (the highest risk) percentiles of the linear predictor from the full model without and with race, respectively.

Figures below show two Kaplan-Meier curves according to risk groups based on the percentiles of the linear predictor [<16th for low-risk group (red), ~16th to <50th for intermediate-risk group (green), ~50th to <84th for higher risk group (blue), and >84th for the highest risk group (purple)] (Figure 2).

The Kaplan-Meier curves of the risk groups were well separated for the high and highest risk groups of the two full models throughout the whole follow-up time. The low-and intermediate-risk groups however became more widely separated by 84 months of follow-up. When comparing our validation results with the ones from the original publication, the discrimination of groups was similar. Furthermore, the full model with race seemed more able to distinguish between the two lowest risk groups in our validation cohort.

The hazard ratios between risk groups were well-maintained. The predicted 5-year risks for patients in the 4 groups defined in our cohort were 27.5, 64.9, 98.4, and 99.9%, respectively for the full model without race, and 35, 73.7, 99.2, and 100%, respectively for the full model with race. Similarly, the eGFR decline slopes in the 4 groups were 1.67, 0.42, 1.18, and 1.77, respectively for the full model with race and 1.23, 0.80, 0.82, and 2.13, respectively for the full model without race. The clinical and histological characteristics of the patients across the risk groups based on the full model without and with race are presented in Table 2. Accordingly, we found that clinical characteristics were worse with increasing risk, defined as higher baseline proteinuria, worst kidney function, and more Oxford MEST lesions.

**Table 2:** Clinical and histological characteristics of groups of patients according to risk stratification based on the full model without and with race.

	**Group 1 (lower 16th percentile)** ***n*** **= 42**	**Group 2 (16–50th percentile)** ***n*** **= 90**	**Group 3 (50–84th percentile)** ***n*** **= 90**	**Group 4 (upper 16th percentile)** ***n*** **= 42**	***P*** **-value**
**Full model without race**
Biopsy age	30 (21.75–42)	39 (30–52.25)	42 (33–52.25)	40.5 (36.5–57.25)	<0.001
Systolic BP	105 (100–116.5)	130 (120–140)	145 (130–150)	157.5 (141.5–167.3)	<0.001
Diastolic BP	69 (60–75)	80 (75–85)	85 (80–90)	90 (80–98)	<0.001
eGFR diagnosis	99.08 (74.99–120.4)	69.35 (52.1–91.9)	50.5 (33.7–67.9)	31 (23.3–46.3)	<0.001
sCr diagnosis	0.85 (0.8–0.9)	1.06 (0.9–1.36)	1.4 (1.1–2.03)	1.95 (1.4–2.63)	<0.001
Proteinuria diagnosis	620 (292.5–1,063)	900 (490–1,500)	1,893 (1,100–3,250)	2,329 (1,788–3,100)	<0.001
M (0/1)	20 (47.6%)/22 (52.4%)	25 (27.8%)/65 (72.2%)	23 (25.6%)/65 (72.2%)	8 (19.1%)/34 (80.9%)	0.023
E (0/1)	36 (85.7%)/6 (42.3%)	63 (70%)/27 (30%)	51 (56.7%)/37 (41.1%)	21 (50%)/21 (50%)	0.002
S (0/1)	29 (69%)/13 (31%)	41 (45.6%)/49 (54.4%)	28 (31.1%)/60 (66.7%)	11 (26.2%)/31 (73.8%)	<0.001
T (0/1/2)	41 (97.6%)/1 (2.4%)/0(0%)	85 (94.4%)/1 (1.1%)/4 (4.4%)	52 (57.8%)/29 (32.2%)/7 (7.8%)	6 (14.3%)/36 (85.7%)/0(0%)	<0.001
**Full model with race**	*n* = 42	*n* = 90	*n* = 90	*n* = 42	
Biopsy age	31.5 (22–42.5)	39 (30–53)	41 (32–52)	45.5 (38–58.25)	0.001
Systolic BP	105 (100–118.5)	128 (120–140)	140 (130–150.5)	154.5 (146.8–167)	<0.001
Diastolic BP	69.5 (60–75)	80 (70–84.5)	85 (80–90)	90 (80.75–98)	<0.001
eGFR diagnosis	96.6 (76.3–114.1)	71 (57.3–92.2)	48.9 (33.7–65.7)	29.3 (21.4–42.9)	<0.001
sCr diagnosis	0.9 (0.8–0.9)	1.02 (0.9–1.2)	1.5 (1.2–1.95)	2.05 (1.65–2.7)	<0.001
Proteinuria diagnosis	500 (252–825)	900 (500–1500)	1800 (1200–2850)	2566 (1800–3937)	<0.001
M (0/1)	17 (40.5%)/25 (59.5%)	28 (31.1%)/61 (67.8%)	25 (27.8%)/64 (71.1%)	3 (7.1%)/36 (85.7%)	0.06
E (0/1)	33 (78.6%)/9 (21.4%)	64 (71.1%)/25 (27.8%)	54 (60%)/35 (38.9%)	20 (47.6%)/22 (52.4%)	0.009
S (0/1)	27 (64.3%)/15 (35.7%)	45 (50%)/44 (48.9%)	28 (31.1%)/61 (67.8%)	9 (21.4%)/33 (78.6%)	<0.001
T (0/1/2)	41 (97.6%)/1 (2.4%)/0 (0%)	84 (93.3%)/1 (1.1%)/4 (4.4%)	53 (58.9%)/29 (32.2%)/7 (7.8%)	6 (14.3%)/36 (85.7%)/0 (0%)	<0.001

*The test used for comparison of continuous variables was ANOVA, and for categorical variables, the Chi-square test was used*.

### Model Calibration

Calibration generally describes the accuracy between the estimation or prediction of survival and the observed survival of the model as seen in the actual data. As previously mentioned by Royston et al. (12) a well-accepted approach to the validation of a model is to estimate the regression coefficient of the prognostic index (PI) in the validation dataset. Here, the PI was first computed for every individual in our cohort exactly as reported for the derivation cohort. Second, the estimate of the calibration slope or the regression coefficient for the PI was calculated for the validation dataset. The overall estimate of the β of the PI is the most precise estimate of the relative global calibration. In our analysis, the estimate of the β_PI_ for the full model with and without race was calculated to be 0.40 (SE = 0.08) and 0.45 (SE = 0.08), respectively, which are far from 1. Thus, it appears that both the models failed to show adequate calibration in this validation cohort and thus cannot accurately predict the 5-year risk for ESKD. This is also apparent in the difference between the predicted mean 5-year risk as calculated from the model (Table 3) and the observed survival as shown in the Kaplan-Meier curves (Figure 2), in which the model overestimates the mean 5-year risk between all risk groups.

**Table 3:** Discrimination measures in the current and reported cohorts.

**Measure**	**Hazard ratio**	**Mean predicted 5y risk, %**	**eGFR decline slope**
**Full model without race**			
Low risk group	Reference	27.5	−1.67
Intermediate risk group	2.15 (0.6–7.6)	64.9	−0.42
High risk group	4.24 (1.2–14.29)	98.4	−1.18
Highest risk group	9.05 (2.6–30)	99.9	−1.77
**Full model with race**			
Low risk group	Reference	35.0	−1.23
Intermediate risk group	1.82 (0.5–6.53)	73.7	−0.80
High risk group	4.55 (1.35–15.26)	99.2	−0.82
Highest risk group	8.66 (2.54–29.5)	100	−2.13

## Discussion

In this study, we validated two risk-prediction models which accurately predict a 50% decline in eGFR or ESKD in patients with IgAN using the available data set from the national IgAN registry of the Greek society of Nephrology (8). From this data set, we extracted and used the clinical and histological information from patients with available MEST scores. In this study, we examined the value and precision in reproducing the predicted risk of a 50% decline in eGFR or ESKD using the already available IgAN international prediction tool from an ethnically homogeneous cohort. Moreover, we examined the validity for both prediction models; the one that includes race/ethnicity in calculating risk and the other without race/ethnicity. Both the models could not accurately capture and predict the 5-year risk; however, they were able to accurately distinguish the highest and higher risk patients from patients with low and intermediate risk.

As the diagnosis of IgAN is only established after a successful renal biopsy containing more than 10 glomeruli, a prediction model with standard histological characteristics would help increase the accuracy of the model. Moreover, there are studies based on urine and serum biomarkers that reflect kidney fibrosis and ongoing disease progression. Nevertheless, the use of such markers has not proven their efficacy in everyday clinical practice (14). Furthermore, the established and histologically reproducible MEST score has a proven value in the long-term prediction of disease progression (15). On the other hand, although some other models for predicting disease progression were developed containing histological variables, these models were either based on a relatively small or in a single patient population (6, 16, 17). In this context, recently two full models combining clinical and histological variables (Oxford MEST score) were derived and validated in two multi-ethnic cohorts (10). These models contain well-established factors for IgAN progression which can easily and consistently be obtained.

Our results point out that both prediction models are fairly suitable for implementation in ethnic Greeks and improve kidney function risk stratification and subsequent decision-making for appropriate clinical treatment. Our analysis showed that survival curves of different risk groups were adequately separated in both the models. Nevertheless, the eGFR decline slope was not consistently larger among the lower, intermediate, and higher risk groups, as it was for the highest risk group. However, both the models showed that the prediction risk over 5 years was extremely overestimated in our patients. Overall, we suggest using the full model without race for further clinical utility assessment and decision-making for pharmacological interventions in the Greek population with IgAN.

The use of immunosuppressive treatment after biopsy in any risk group of our cohort reached 46.2% which is higher than that used in the originally reported validation cohort (10) as well as in another Asian-Caucasian cohort which was used for external validation of the risk-prediction model (11). Moreover, 26.2% in the lowest and 33.3% of patients in the intermediated risk group received immunosuppressive treatment as well. This is in accordance with other studies which point that a large proportion of up to 75% of patients are over-treated with immunosuppression despite having a non-progressive disease even when its value has not been proved in large prospective randomized trials (18–21). On the contrary, the majority, but not all of the patients that showed higher or the highest risk of disease progression received immunosuppressive treatment consisting either of corticosteroids alone (per o.s. or i.v.) or with a combination with either azathioprine or cyclophosphamide. Although the exact risk stratification threshold for immunosuppression initiation is yet to be determined based on the risk-benefit ratio, having a reliable, easy-to-use tool, will facilitate clinical trial design by focusing on different treatment regimens according to the individualized patient risk of disease progression. Accordingly, this will eventually offer and configurate the appropriate risk-based treatment protocols.

Although the IgAN risk tool was evaluated in an international, multiethnic cohort addressing issues of previous studies, such as small size cohorts with only a few patients across the spectrum of disease activity and inter-ethnicity difference, we believe that our cohort further enhances the validity of previous results. That is because our cohort consists of an adequate number of patients that covers the whole spectrum of disease activity and with a long follow-up of more than 8 years. Furthermore, as the original prediction risk tool accommodates differences across different ethnic groups, we believe that the use of our group of patients highlights not only specific similarities but also disparities compared to the international population (7).

Our patients showed a similar burden of total primary outcome events in comparison to both the originally reported validation cohort (10) as well as in another Asian-Caucasian cohort (11). Nevertheless, the total follow-up of these events that were captured was significantly higher in our cohort. This was despite a significantly lower median eGFR at diagnosis and a higher proportion of patients with established chronic kidney disease stage III or worst. Furthermore, this was not accompanied by significant differences in conservative renin-angiotensin-aldosterone system inhibitor (RAASi) treatment trends after diagnosis, in comparison to the other validation cohorts (10, 11). In comparison to the original validation cohort (10), RAASi initiation before diagnosis was higher but in comparison to the cohort by Zhang et al. (11), it was significantly lower. This was probably due to different treatment approaches in patients of the current and older eras that were included in our cohort. In any case, this strengthens the analysis as our cohort represents both current and older treatment regimens. Concerning immunosuppression, a slightly higher percentage of our patients received such treatment in comparison to both previously mentioned validation cohorts. Thus, the unexpected better 5-year survival of our cohort could in part be attributed not only to different treatment decisions and trends but also highlights the differences in disease progression among ethnic groups (22).

The strength of our study is that it used a population of patients with long-term follow-up, far more than the original and other validation cohorts (7, 23). This gives us the advantage of capturing those patients with silent and gradual but ominous disease progression well beyond after diagnosis which is an IgAN characteristic (24). However, a limitation of our study is the exclusion from the final cohort of those patients who did not have an available Oxford MEST histologic score. This group of patients is currently the largest in the IgAN registry of the Greek society of Nephrology which means that we have missed some intermediate-risk patients who showed a gradual disease progression. Moreover, Group 2 and Group 3 have 4 and 7 patients, each with T2 lesions in biopsy thus highlighting significant interstitial fibrosis and tubular atrophy while Group 4 does not have a patient with T2 lesion (please refer to Table 2), nevertheless, the percentage of patients in total with T2 lesions in our cohort is identical to that of the original derivation cohort (4.6 vs. 4.2% in our cohort); thus, we consider that this finding cannot compromise our results. Another limitation of the prediction model is that it can be used only for a relatively short-term prognosis (up to 8 years), considering that IgAN has a long-term evolution.

In conclusion, we validated the full prediction models for risk stratification of patients with IgAN. These models showed an inferior performance on a personalized risk assessment in comparison to one of the original derivation cohorts. Specifically, this tool can precisely stratify Greek patients with IgAN into four major risk groups (low, intermediate, high, and highest risk) but without accurately predicting their 5-year kidney function. Overall, this tool may help discriminate high-risk patients who will benefit from immunosuppression treatment and avoid such interventions in those with low risk for disease progression. However, it is important to re-validate this tool in a larger population to further investigate its accuracy which emphasizes the need to expand the Greek national and other international IgAN registries.

## Data Availability Statement

The original contributions presented in the study are included in the article/Supplementary Material, further inquiries can be directed to the corresponding author/s.

## Ethics Statement

The studies involving human participants were reviewed and approved by University Hospital of Patras. The patients/participants provided their written informed consent to participate in this study.

## Author Contributions

DG and MP: conceptualization. GS and MP: methodology. DC: software. DC and GS: validation. DC, GS, and MP: formal analysis. HG and GL: investigation. SM, DX, ES, GL, EK, SZ, MK, GM, SF, ED, AD, APd, ND, DB, and KS: resources and data curation. MP: writing—original draft preparation. MP, MS, and DC: writing—review and editing. MP and EP: visualization. EP, IB, and APg: supervision. DG: project administration. All authors contributed to the article and approved the submitted version.

## Funding

This work was supported by a grant from the Hellenic Society of Nephrology.

## Conflict of Interest

The authors declare that the research was conducted in the absence of any commercial or financial relationships that could be construed as a potential conflict of interest.

## Publisher's Note

All claims expressed in this article are solely those of the authors and do not necessarily represent those of their affiliated organizations, or those of the publisher, the editors and the reviewers. Any product that may be evaluated in this article, or claim that may be made by its manufacturer, is not guaranteed or endorsed by the publisher.

## Supplementary Material

Supplementary Figure 1 Representative mesangial [M] and endocapillary [E] hypercellularity, segmental sclerosis [S], and interstitial fibrosis/tubular atrophy [T] (MEST) score kidney biopsy pictures. **(A)** MEST: M1; Glomerulus with global severe mesangial hypercellularity, HE x 400. **(B)** MEST: E1; Mesangial hypercellularity and segmental endocapillary hypercellularity, PAS x 400. **(C)** MEST: S1; Segmental glomerulosclerosis with adhesion to Bowman's capsule that lies closer to the vascular pole (arrow), HE x 400. **(D)** MEST: S1; Segmental glomerulosclerosis with adhesion to Bowman's capsule that lies close to the tubular pole (arrow), Masson x 400. **(E)** MEST: T0; Minimal Interstitial fibrosis in a patient with IgA nephropathy, Masson x 200. **(F)** MEST: T1; Focus of tubular atrophy and interstitial fibrosis in a patient with IgA nephropathy whose biopsy showed moderate but no more than 50% tubular atrophy or interstitial fibrosis (arrow), PAS x 100. **(G)** MEST: T2; Interstitial fibrosis and tubular atrophy in a patient with IgA nephropathy whose biopsy showed diffuse interstitial fibrosis, Masson x 200. **(H)** MEST: C1; Endocapillary hypercellularity with crescent formation (arrow), HE x 400. HE, hematoxylin and eosin staining; PAS, periodic acid-Schiff staining.
