# Supplementary figures and images for: Validation of the International IgA Nephropathy Prediction Tool in the Greek Registry of IgA Nephropathy
Source: Front Med (Lausanne). 2022 Feb 15;9:778464. doi: 10.3389/fmed.2022.778464 (PMC8885590; doi:10.3389/fmed.2022.778464)

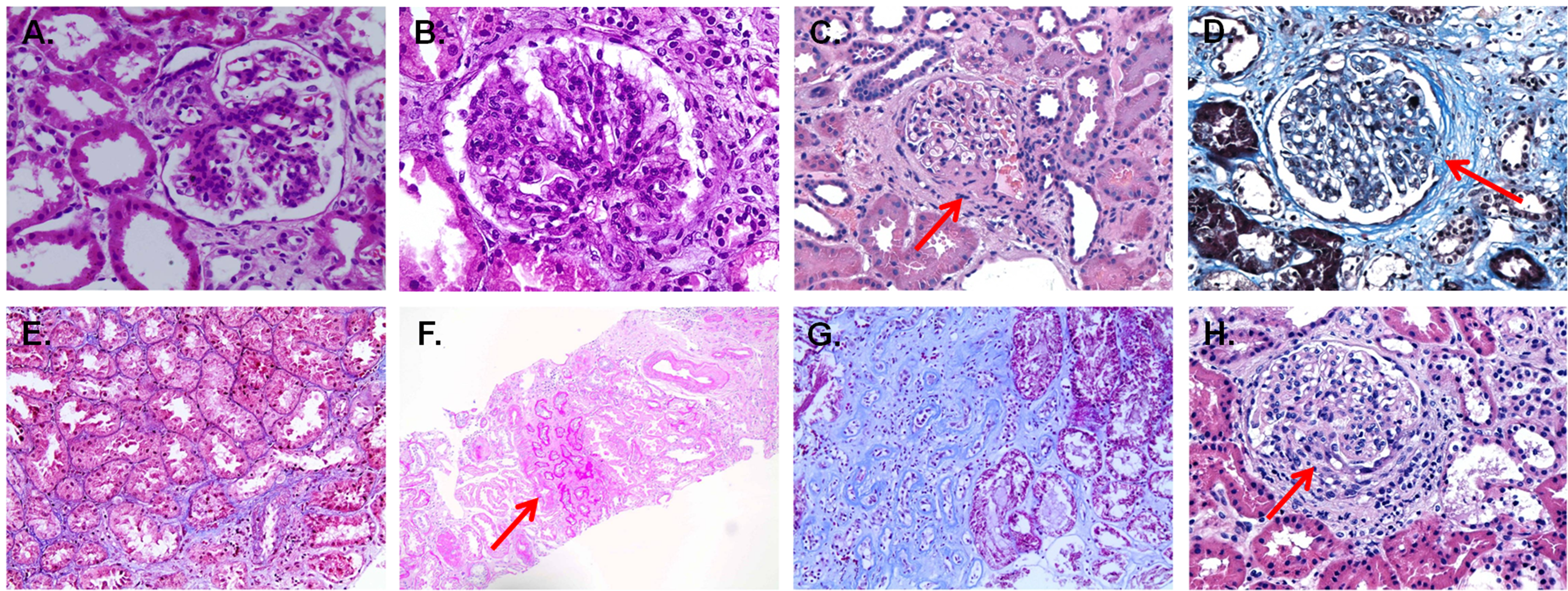

Supplement: Supplementary Figure 1 — Representative mesangial [M] and endocapillary [E] hypercellularity, segmental sclerosis [S], and interstitial fibrosis/tubular atrophy [T] (MEST) score kidney biopsy pictures. (A) MEST: M1; Glomerulus with global severe mesangial hypercellularity, HE x 400. (B) MEST: E1; Mesangial hypercellularity and segmental endocapillary hypercellularity, PAS x 400. (C) MEST: S1; Segmental glomerulosclerosis with adhesion to Bowman's capsule that lies closer to the vascular pole (arrow), HE x 400. (D) MEST: S1; Segmental glomerulosclerosis with adhesion to Bowman's capsule that lies close to the tubular pole (arrow), Masson x 400. (E) MEST: T0; Minimal Interstitial fibrosis in a patient with IgA nephropathy, Masson x 200. (F) MEST: T1; Focus of tubular atrophy and interstitial fibrosis in a patient with IgA nephropathy whose biopsy showed moderate but no more than 50% tubular atrophy or interstitial fibrosis (arrow), PAS x 100. (G) MEST: T2; Interstitial fibrosis and tubular atrophy in a patient with IgA nephropathy whose biopsy showed diffuse interstitial fibrosis, Masson x 200. (H) MEST: C1; Endocapillary hypercellularity with crescent formation (arrow), HE x 400. HE, hematoxylin and eosin staining; PAS, periodic acid-Schiff staining. [file Image_1.PNG]
